# Induction of Cryptic and Bioactive Metabolites through Natural Dietary Components in an Endophytic Fungus *Colletotrichum gloeosporioides* (Penz.) Sacc.

**DOI:** 10.3389/fmicb.2017.01126

**Published:** 2017-06-19

**Authors:** Vijay K. Sharma, Jitendra Kumar, Dheeraj K. Singh, Ashish Mishra, Satish K. Verma, Surendra K. Gond, Anuj Kumar, Namrata Singh, Ravindra N. Kharwar

**Affiliations:** ^1^Mycopathology and Microbial Technology Laboratory, Centre of Advanced Study in Botany, Department of Botany, Institute of Science, Banaras Hindu UniversityVaranasi, India; ^2^Botany Section, Mahila Maha Vidyalaya, Banaras Hindu UniversityVaranasi, India; ^3^Department of Botany, Buddha PG CollegeKushinagar, India

**Keywords:** endophytic fungi, epigenetic modulation, HPLC, metabolites, *Syzygium cumini*, *Colletotrichum gloeosporioides*

## Abstract

Grape skin and turmeric extracts having the major components resveratrol and curcumin, respectively, were used for the induction of cryptic and bioactive metabolites in an endophytic fungus *Colletotrichum gloeosporioides* isolated from *Syzygium cumini.* The increase in total amount of crude compounds in grape skin and turmeric extract treated cultures was 272.48 and 174.32%, respectively, compared to the untreated control. Among six human pathogenic bacteria tested, the maximum inhibitory activity was found against *Aeromonas hydrophila* IMS/GN11 while no inhibitory activity was observed against *Enterococcus faecalis* IMS/GN7. The crude compounds derived from turmeric extract treated cultures showed the highest DPPH free radicals scavenging activity (86.46% inhibition) followed by compounds from grape skin treated cultures (11.80% inhibition) and the control cultures (1.92% inhibition). Both the treatments significantly (*p* ≤ 0.05) increased the antibacterial and antioxidant activities of crude metabolites compared to the control. HPLC profiling of crude compounds derived from grape skin and turmeric extract treated cultures revealed the presence of additional 20 and 14 cryptic compounds, respectively, compared to the control. These findings advocate the future use of such dietary components in induced production of cryptic and bioactive metabolites.

## Introduction

The term endophytes was coined by the German scientist de Bary (1866) for any organism (bacteria, fungi, actinomycetes, etc.) that inhabit healthy plant tissues internally, without causing any identifiable disease symptoms to the host. Normally, endophytes obtain nutrients and protection from their host and in turn significantly contribute to enhance the defense mechanism of host against pathogens, herbivores, and abiotic stresses. Until 1990, endophyte researches, were mainly confined to the diversity, distribution, ecology and host health, but a new avenue opens up and got the momentum after the synthesis of host mimetic billion dollar anticancer compound taxol by *Taxomyces andreanae*, an endophytic fungus of *Taxus brevifolia* (Stierle et al., 1993; Kharwar et al., 2011). The success of obtaining fungal taxol has initiated a paradigm for the search of still other bioactive compounds to be found in endophytic microbes. Later several other host mimetic compounds were reported from endophytic fungi such as, camptothecine, vincristine, vinblastine, rohitukine, azadirachtine, and piperine. Such alternative potential fungal sources may reduce the prices of host mimetic compounds and over exploitation of host plants (Verma et al., 2009; Kusari et al., 2012; Chithra et al., 2014; Su et al., 2014; Monggoot et al., 2017). Endophytic fungi are also well known producer of other potential natural compounds of agricultural, industrial and pharmaceutical interest. Some recent reviews have thoroughly discussed the considerable numbers of antibacterial compounds isolated from endophytic microbes and their efficacy and probable usages (Mishra et al., 2012, 2016; Deshmukh et al., 2015; Rao et al., 2017). More than 100 anticancer compounds have been reported alone from fungal endophytes between the period of 1990–2010 (Kharwar et al., 2011).

*Colletotrichum gloeosporioides* (Penz.) Sacc., is known to produce several biologically active compounds such as 10-hydroxy camptothecine, aspergillomarasmine A and B and highly antimicrobial gloeosporone. This fungus also produces some host mimetic anticancer compounds like taxol, piperine, and rohitukine (Senthilkumar et al., 2013; Chithra et al., 2014; Kumara et al., 2014; Su et al., 2014). One novel compound viz. 2-phenylethyl 1*H*-indol-3-yl-acetate, and seven known compounds viz. uracil, cyclo-(*S*^∗^-Pro-*S*^∗^-Tyr), cyclo-(*S*^∗^-Pro-*S*^∗^-Val), 2(2-aminophenyl) acetic acid, 2(4-hydroxyphenyl) acetic acid, 4-hydroxy-benzamide, and 2(2-hydroxyphenyl) acetic acid with antifungal, anticancer and acetylcholinesterase (AChE) inhibitory activities have also been reported from *C. gloeosporioides* (Chapla et al., 2014). Gloeosporone, a self conidial germination inhibitory compound and ferricrocin, a phytotoxin, have been isolated from *C. gloeosporioides* (Meyer et al., 1983; Ohra et al., 1995). Highly functional antibacterial compounds azaphilones and colletotric acid and a ring B aromatic steroid are also known to be produced by endophytic *C. gloeosporioides* (Zhang et al., 2009; Wang et al., 2016).

*Syzygium cumini* (Syn. *Eugenia jambolana* Lam.) has great economic importance as its most parts like bark, leaf, seed, and fruits are used in alternative medicine to treat various diseases. This plant was selected to isolate the endophytic fungi in anticipation of receiving the potential strain because of its various medicinal properties like antimicrobial, antiviral, anti-genotoxic, anti-inflammatory, anti-ulcerogenic, anti-allergic, cardio protective, anticancer, chemo preventive, radio protective, antioxidant, hepatoprotective, anti-diarrheal, hypoglycemic, and anti-diabetic activities (Baliga et al., 2011). *S. cumini* also contains various important phytochemicals like tannins, terpenoids, gallic acid, glycoside jambolin, anthocyanins, and various minerals (Chaudhary and Mukhopadhyay, 2012). In filamentous fungi, the biosynthetic genes for secondary metabolites are typically arranged in cluster (Keller and Hohn, 1997; Walton, 2000). Generally, in optimum laboratory conditions, many gene clusters responsible for the biosynthesis of secondary metabolites remains silent and cryptic (Rutledge and Challis, 2015). These cryptic or silent genes can also be induced through epigenetic modulations. A recent review reported that the endophytes can produce even more compounds with far greater potential by using epigenetic modulations (Fischer et al., 2016). Epigenetics is the study of changes in the expression and regulation of the genes that are not dependent on DNA sequences. The idea of epigenetics was introduced by Waddington (1942) for the development of specific traits by interaction of genes and its environmental factors. The two broad mechanisms of epigenetic modulation are the DNA methylation and histone modifications. The fungal treatment with DNA methyltransferase (DNMT) inhibitors like 5-azacytidine, 5-aza-2′-deoxycytidine, hydralazine, procaine, procainamide and/or histone deacetylase (HDAC) inhibitors like sodium butyrate, suberoylanilide hydroxamic acid (SAHA), valproic acid are effective in facilitating the activation of biosynthetic pathways of secondary metabolites that are dormant under regular conditions. However, some recent studies have suggested that various components of dietary items like turmeric, grapes, green tea, soybean, and cruciferous vegetables can also bring epigenetic changes (vel Szic et al., 2010; Hardy and Tollefsbol, 2011; Abdulla et al., 2013). These bioactive dietary components alter the DNA methylation and histone modifications requisite for gene activation the same way as the chemical epigenetic modifiers (Meeran et al., 2010). Folate, methionine, betaine, choline, and vitamin B-12, alters the 1-carbon metabolism that influences DNA and histone methylation (Choi and Friso, 2010). Catechins, the phenolic compounds of natural origin found in tea, are effective inhibitors of human DNMT-mediated DNA methylation in cultured cancer cells (Fang et al., 2003). Curcumin, a component of *Curcuma longa* (turmeric), has recently been determined to induce epigenetic changes by regulating histone acetyltransferases, histone deacetylases DNA methyltransferase I, and miRNAs activities (Reuter et al., 2011). Epigenetic influences of intake of the dietary nutrients and bioactive food components have been much studied in the field of environmental and cancer research (Su et al., 2012). But the influence of natural dietary components on the induction of secondary metabolites is not studied till date. Considering the above facts present study has been designed to assess the effects of grape skin and turmeric extract on the production of cryptic and antimicrobial rich compounds by endophytic fungus *C. gloeosporioides* (Penz.) Sacc.

## Materials and Methods

### Plant Sample Collection, Surface Sterilization, Isolation and Identification of Endophytic Fungi

Mature healthy, asymptomatic leaves were collected from the tree of *Syzygium cumini* from botanical garden of BHU (Banaras Hindu University) campus, Varanasi. The collected leaves were placed in polythene bags and were stored at 4°C. The leaf samples were washed thoroughly in running tap water for 30 min and then rinsed with double distilled water to remove the debris adhered. Methodology given by Petrini et al. (1992) was adopted for the surface sterilization of leaves and its effectiveness was checked following leaf imprint method (Schulz et al., 1993). Epiphytic microbes were eradicated by immersing the tissues in 90% ethanol for ∼1 min and in aqueous sodium hypo chlorite solution (2% available chlorine) for ∼2 min, followed by washing with 70% ethanol for ∼10 s. The leaf tissues were then rinsed in sterile distilled water and allowed to surface dry in sterile condition. After surface treatment the samples were then carefully dissected into small pieces of approximately 0.5 mm^2^ × 0.5 mm^2^ size. The 4–5 small pieces were placed in Petri dishes containing potato dextrose agar (PDA) medium supplemented with streptomycin (250 mg/l) and were incubated for 21 days at 26 ± 2°C in a BOD cum humidity incubator (Caltan Super Deluxe, NSW, New Delhi).

### Molecular Characterization of Endophytic Fungal Isolate

Genomic DNA of the endophytic fungus *C. gloeosporioides* was extracted following the modified protocol of Sim et al. (2010). The universal primers ITS-1: 5′TCCGTAGGTGAACCTGCGG3′ and ITS-4: 5′TCCTCCGCTTATTGATATGC3′ (Metabion International, Martinsried, Germany), were used to amplify the 5.8S rDNA and two ITS regions between 18S and 28S rDNA. Total PCR mixture of 25 μl, containing 1 μl (100 ng/μl) DNA template, 1 μl each primer, 0.33 μl (3 units/μl) *Taq* polymerase, 0.5 μl dNTPs, 2.5 μl 10× PCR buffer with 25 mM MgCl_2_ and 18.67 μl milli Q water was used for the PCR. PCR was performed in Mycycler (BioRad, Hercules, CA, United States) under the following conditions: pre-denaturation at 94°C for 4 min; 35 cycles of each denaturation at 94°C for 1 min, annealing at 55°C for 1 min, extension at 72°C for 1 min; and then a final extension at 72°C for 5 min. Amplified PCR products were resolved by electrophoresis on a 1.5% (w/v) agarose gel stained with ethidium bromide (0.5 μg/ml) for visual examination. PCR amplified DNA was purified using HiYield PCR DNA mini kit from Real Biotech Corporation (RBC, India) through gel excision method. Purified DNA was sequenced by Amnion Biosciences Pvt. Ltd, India. The ITS rDNA sequences obtained were used to retrieve similar sequences from the NCBI GenBank sequence database using the NCBI nBLAST program. The rDNA sequence was submitted to NCBI GenBank database for identification and accession number.

### Preparation of Extracts from Turmeric and Grape Skin for Epigenetic Treatment

One gram of each grape skin and turmeric was separately crushed in 100 ml methanol with the help of mortar and pestle and left overnight at 4°C followed by filtration with Whatman no. 1 filter paper. Filtrates were vacuum evaporated with the help of rotary evaporator (IKA, Germany). Dried crude extract were weighted and dissolved in a concentration of 10 mg/ml in milli Q water as stock solutions, and were then filtered with 0.20 μm filter paper for removal of any contaminant. PDB medium amended with 10 μg/ml of crude extract in each case was prepared. Finally test culture was inoculated and incubated at 26 ± 2°C. The treatments were done in triplicate for reproducibility.

### Isolation of Secondary Metabolites

After 21 days of incubation, the broth cultures were filtered by Whatman filter paper and filtrates were subjected to extraction thrice with the equal volume of ethyl acetate. The extracted secondary metabolites were concentrated and evaporated by rotary vacuum evaporator (IKA, Germany). The total amount of the secondary metabolites obtained from different treatments were measured and dissolved in methanol to the final concentration of 1 mg/5 μl.

### Antibacterial Activity

The metabolites, extracted after different treatments of endophytic fungi, were screened for antibacterial activity against the six human bacterial pathogens using disk diffusion method. To the sterilized filter paper disks, 5 μl (1 mg) of crude extract was loaded aseptically and air dried. Antibacterial test was performed against the following gram positive and gram negative bacteria – *Aeromonas hydrophila* IMS/GN11, *Shigella boydii* IMS/GN17, *Salmonella typhi* MTCC 3216, *Staphylococcus aureus* ATCC 25923, *Escherichia coli* IMS/GN19, and *Enterococcus faecalis* IMS/GN7. The lawn of test bacterial culture was prepared with cotton swab on the surface of solidified Mueller-Hinton agar Petri plates. The paper disks containing 1 mg crude extract were placed on the surface of the Mueller-Hinton medium seeded with test bacterium in Petri plate. The paper disk dried after impregnating 5 μl methanol was placed as positive control. Plates were incubated for 24 h at 35 ± 2°C and then were analyzed for antibacterial activity by observing the zone of inhibition. Each test was done in triplicates and the means of the diameter of the inhibition zones with standard deviation were presented in the results.

### Antioxidant Assay

The scavenging activity of the secondary metabolites of different treatments and control was measured on DPPH radicals following the method devised by Shen et al. (2010) with minor modifications. An aliquot of 1 ml of 0.2 mM DPPH radical (Sigma) in methanol was added to a test tube containing 3 ml of methanolic solution of crude extract (1 mg/3 ml). The reaction mixture was vortex mixed at room temperature and the absorbance (Abs) was taken at 517 nm after 30 min of incubation at room temperature in the dark. The ascorbic acid was used as standard antioxidant and the methanol as control. The % inhibition was calculated against control as [(Abs_control_ - Abs_test sample_) ÷ Abs_control_ × 100].

All the data were statistically analyzed by analysis of variance (ANOVA) and means were compared by Tukey’s honestly significant differences (HSD) test. All the analyses were performed using Graph pad prism 5.1 software.

### HPLC Profiling of Crude Compounds

HPLC analysis of crude compounds was done on RP-C18 column using Photodiode Array Detectors (PDA). The injection volume and flow rate used were 20 μl and 0.50 ml/min, respectively. Acetonitrile along with double distilled water were used as mobile solvent. Elution program of compounds started with 15% acetonitrile reaching up to 100% in 40 min with a hold on this condition for 5 min, and again gradient coming down to 15% acetonitrile in 8 min which was finally held for 5 min. The samples and mobile phase were filtered through 0.2 μm nylon membrane filter before applying into the column. All samples were analyzed at 254 nm wavelength.

## Results

### Isolation and Identification of Endophytic *Colletotrichum gloeosporioides*

**FIGURE 1:**
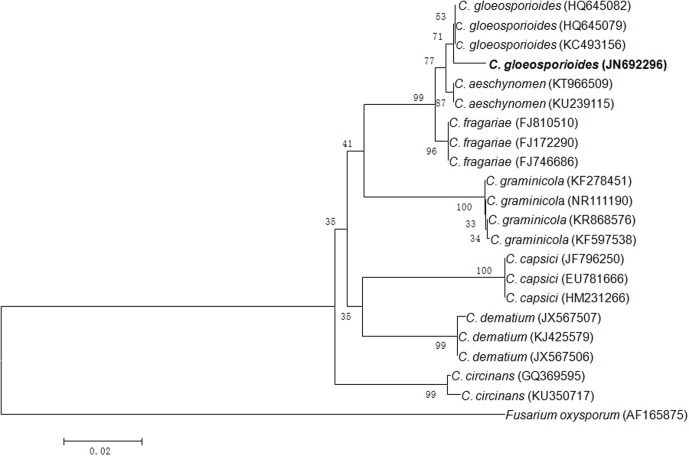
Neighbor-joining tree from ITS sequences showing the relationship between *Colletotrichum* species of the present study and other closely related *Colletotrichum* species retrieved from the GenBank. Bootstrap values (1000 replicates) are shown on the branches. Bar = 2 nucleotide substitutions per 100 nucleotides.

In present study, an endophytic fungus was isolated from the surface sterilized leaves of *Syzygium cumini* growing in the botanical garden of BHU campus, Varanasi. On the basis of ITS rDNA gene sequencing, it was identified as *C. gloeosporioides* with GenBank accession number JN692296 (**Figure 1**).

### Crude Metabolites and Antibacterial Activity of Treated Cultures

The amount of crude secondary metabolite extracted was highest (406 ± 3.46 mg/500 ml of broth) from the cultures of *C. gloeosporioides* treated with grape skin extract followed by the cultures treated with turmeric extract (299 ± 9.07 mg/500 ml of broth) and the lowest (109 ± 9.54 mg/500 ml of broth) in the control cultures. Thus, in contrast to control the amount of secondary metabolites secreted was 272.48 and 174.32% more in the cultures treated with grape skin and turmeric extracts, respectively.

**FIGURE 2:**
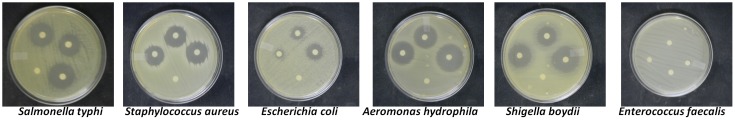
Antibacterial activity of crude compounds isolated from treated and untreated control cultures of *Colletotrichum gloeosporioides*.

The crude compounds when screened for antibacterial activity against six human pathogenic bacteria showed a high antibacterial activity against *A. hydrophila* IMS/GN11, *Shigella boydii* IMS/GN17, *Salmonella typhi* MTCC 3216, *Staphylococcus aureus* ATCC 25923, and *Escherichia coli* IMS/GN19 (**Figure 2** and **Table 1**). Contrarily, *Enterococcus faecalis* IMS/GN7, was not at all susceptible to the secondary metabolites of the any treatment or control. The zone of inhibition against *A. hydrophila* was found maximum (27 mm) by the metabolites extracted from the grape skin treated culture followed by turmeric extract treated culture (25 mm) and the control culture (23 mm). Similar trend in the zone of inhibition was also recorded against *S. boydii, S. typhi, S. aureus*, and *E. coli* for the metabolites extracted from the cultures treated with grape skin extract, turmeric extract and control (**Figure 2** and **Table 1**). Both the treatments showed significant (*p* ≤ 0.05) increase in antibacterial activity of metabolites compared to control.

**Table 1:** Antibacterial activity of crude metabolites of different treatments.

Treatments	Inhibition zone (mm)					
	*Staphylococcus aureus*	*Enterococcus faecalis*	*Shigella boydii*	*Salmonella typhi*	*Aeromonas hydrophila*	*Escherichia coli*
Control	19.0 ± 1.7^c^	0 ± 0^a^	18.3 ± 0.6^c^	19.0 ± 2.0^b^	23.0 ± 1.0^b^	8.0 ± 0.0^c^
Grape skin extract	20.3 ± 0.6^bc^	0 ± 0^a^	25.3 ± 0.6^a^	22.0 ± 1.7^a^	27.3 ± 1.5^a^	16.3 ± 0.6^a^
Turmeric extract	22.0 ± 1.0^a^	0 ± 0^a^	22.0 ± 1.7^b^	20.0 ± 1.0^ab^	25.3 ± 0.6^ab^	11.7 ± 1.2^b^

*Values (Mean ± SD) sharing a common letter within the column is not significant at *p* ≤ 0.05*.

### Antioxidant Assay

**FIGURE 3:**
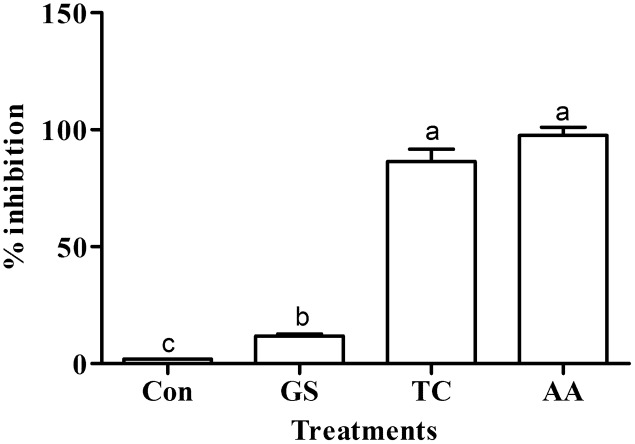
Effect of grape skin (GS) and turmeric (TC) extracts on the antioxidant activity. Error bars represent ± Standard deviation. AA represents ascorbic acid (100 μg ml^-1^) used as a positive control. Bars sharing a common letter(s) are not significant at *P* < 0.05.

The scavenging activity of different crude metabolites against the DPPH radical in terms of % inhibition is given in **Figure 3**. Like the standard ascorbic acid antioxidant activity (97.85% inhibition), the near similar antioxidant activity (86.46% inhibition) was observed by the secondary metabolites of the culture treated with turmeric extract. However, metabolites from the culture treated with grape skin extract showed lower antioxidant activity (11.80% inhibition) even though it was multifold higher than the untreated control culture (1.92% inhibition). Antioxidant activity of treatments significantly differed (*p* ≤ 0.05) with one another and that of the control (**Figure 3**).

### HPLC Analysis of Crude Compounds

**FIGURE 4:**
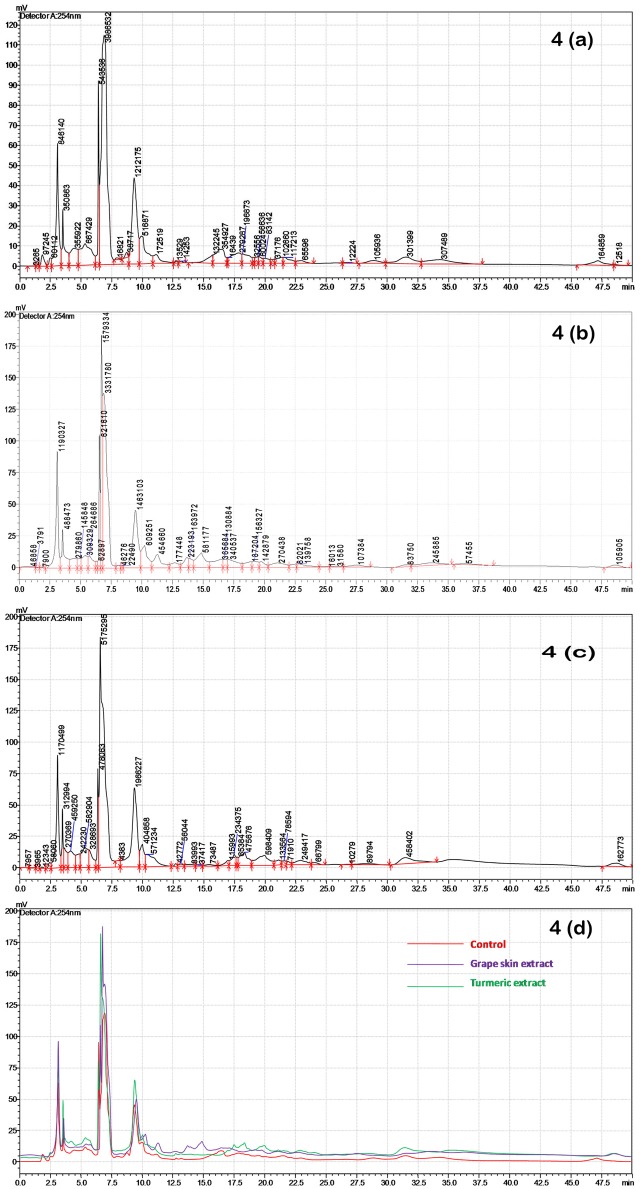
HPLC profiles of secondary metabolites isolated after grape skin and turmeric extract treatments of *C*. *gloeosporioides* at 254 nm; **(a)** HPLC chromatogram of the untreated control; **(b)** HPLC chromatogram of grape skin extract; **(c)** HPLC chromatogram of turmeric extract; **(d)** overlay of control, grape skin extract and turmeric extract.

HPLC analysis of the crude compounds derived from differently treated cultures revealed that the treatment of *C. gloeosporioides* by grape skin and turmeric extracts activated the secretion of many cryptic compounds that were not observed in the untreated samples (**Figures 4a–d** and Supplementary Table 1). This analysis clearly demonstrated that the crude secondary metabolites of the grape skin treated culture produced 37 compounds while turmeric extract treated culture showed 35 compounds and the untreated control culture displayed 34 compounds. The number of cryptic compounds was more in the secondary metabolites extracted from the culture treated with the grape skin extract (20) as compared to those in the culture treated with turmeric extract (14). Although in the treated cultures many cryptic compounds were encountered, but at the same time certain compounds which were present in the control were found missing in the treated ones. The number of common compounds present in the treated as well as control samples were detected more in the culture treated with turmeric extract (21) as compared to those (17) in the culture treated with the grape skin extract (**Table 2**).

**Table 2:** Different category of compounds based on the HPLC profile of the total metabolite of *Colletotrichum gloeosporioides* from different treatments.

	Control	Grape skin extract	Turmeric extract
Total number of compounds detected	34	37	35
Number of common compounds detected in treated and control		17	21
Number of cryptic compounds detected in treated cultures		20	14
Number of compounds missing in treated cultures		17	13

## Discussion

Initially most of the epigenetic studies, were confined to the developmental and clinical biology particularly for the treatment of various life threatening diseases, but in the last few decades, researchers from other fields of biology, including the endophytic microbiology, also showed interest in this fascinating area of research (Brueckner and Lyko, 2004; Feinberg and Tycko, 2004; Chen et al., 2013; Kumar et al., 2016). *Colletotrichum*, the present study material was isolated from the healthy leaf segments of *Syzygium cumini. C. gloeosporioides* has also been reported from a vast range of host plants including *Artemisia mongolica, Artemisia annua, Justicia gendarussa, Theobroma cacao, Vitex negundo*, and *Buxus sinica* (Lu et al., 2000; Zou et al., 2000; Gangadevi and Muthumary, 2008; Mejía et al., 2008; Arivudainambi et al., 2011; Wang et al., 2016).

In this study, the secondary metabolites of *C. gloeosporioides*, extracted with ethyl acetate showed significant antibacterial activity against five human pathogenic bacteria *viz. A. hydrophila*, *S. boydii*, *S. typhi*, *S. aureus*, and *E. coli.* No inhibitory activity was registered against *E. faecalis* (**Figure 2** and **Table 1**). A number of bioactive compounds have been reported from *C. gloeosporioides s*upporting our finding. Previously, *C. gloeosporioides*, an endophytic fungus isolated from the stem of *Artemisia mongolica*, was found to produce new antimicrobial metabolite, named colletotric acid in broth culture (Zou et al., 2000). In another report endophytic *Colletotrichum* sp., isolated from *Artemisia annua* stem was shown to produce three novel and seven known compounds, out of which all novel and only three known compounds exhibited antibacterial activity against tested bacteria (Lu et al., 2000). The metabolites of endophytic *C. gloeosporioides* were also reported to have anticancer activity. *C. gloeosporioides* (JGC-9) isolated from *Justicia gendarussa*, was reported to secrete 163.4 μg/L of taxol in liquid culture which showed strong cytotoxic activity toward BT 220, H116, Int 407, HL 251, and HLK 210 human cancer cells *in vitro* (Gangadevi and Muthumary, 2008). The treatment of *Theobroma cacao* with its own endophyte *C. gloeosporioides* significantly reduced pod loss due to *Moniliophthora roreri* (frosty pod rot) and *Phytophthora palmivora* (black pod rot) in field trials, suggesting the biocontrol potential of fungal endophytes (Mejía et al., 2008). Novel bioactive metabolites from this endophytic fungus isolated from the medicinal plant *Vitex negundo* L. registered effective antimicrobial activity together with strong activity against multidrug-resistant *Staphylococcus aureus* indicating that these metabolites can be a potential source of new antibiotics (Arivudainambi et al., 2011). In a recent study an endophytic fungus, *Colletotrichum* sp. (BS4), isolated from the leaves of *Buxus sinica* was reported to produce three novel compounds, colletotrichones A–C, and one known compound, chermesinone B having antibacterial properties (Wang et al., 2016).

The cultures treated with the grape skin and turmeric extracts showed increased antibacterial activity against the tested human bacterial pathogens. Resveratrol is main component of the grape skin extract while curcumin is the main component of turmeric extract. Both of these compounds are known to have potential to bring epigenetic changes required for gene expression. Resveratrol, a polyphenolic compound commonly found in grapes, berries, and peanuts, is used in the epigenetic alteration for gene expression for therapeutic uses. But the use of resveratrol in epigenetic activation of the silent gene clusters for secondary metabolites production in fungi is not common. Resveratrol prevents epigenetic silencing of BRCA-1 protein which is tumor suppressor and is involved in the repair of DNA damage through aromatic hydrocarbon receptor in human breast cancer cells (Papoutsis et al., 2010). Resveratrol, which binds and activates estrogen receptors, are important factor in the regulation of transcription of estrogen-responsive target genes and increases expression of BRCA1 and BRCA2 mRNA in breast tumor cell lines (Fustier et al., 2003). Resveratrol also increases the expression of native estrogen-regulated genes, and stimulates the proliferation of estrogen-dependent T47D breast cancer cells. It also acts as an agonist for the estrogen receptor (Gehm et al., 1997). Just like resveratrol, the curcumin is also not frequently used in the epigenetic induction of silent genes for cryptic metabolites in fungal systems. Curcumin is also a polyphenolic compound that is well known as an inhibitor of DNA methyltransferase, beside its interaction together with microRNAs, reestablishes the balance between histone acetyl transferase and HDAC activity to selectively regulate the expression of genes concerned with human cancer (Fu and Kurzrock, 2010; Teiten et al., 2013). Wilken et al. (2011) discussed the anticancer property of the curcumin.

Through HPLC we have recorded the increase in the number of cryptic metabolites after successful induction by the grape skin and turmeric extract (**Figure 4**). The increased number of cryptic compounds can be related with the enhanced antibacterial activity of the secondary metabolites isolated from the treated cultures of an endosymbiotic fungus *C. gloeosporioides* (**Table 2**). Though some compounds were found missing in the treated cultures, in most probability, due to epigenetic silencing of gene(s) associated with the biosynthetic pathways of these compounds, the basic aim of the present study to increase the number of compounds with improved bioactivity by treating the cultures with the grape skin and turmeric extracts seems to be fulfilled with significant (*p* ≤ 0.05) increase in DPPH radical scavenging and antibacterial activities (**Figure 3**).

Epigenetic modulations in the endophytic fungus not only improve the production of cryptic metabolites, but it also opens a new possibility for the regulation of secondary metabolites synthesis and the illustration of metabolite pathways of the cryptic natural compounds. In a recent study an endophytic actinomycetes, *Streptomyces coelicolor* strain AZRA 37 horbouring *Azadirachta indica* A. Juss., when treated with 5-azacytidine (25 μM) increased the number of compounds in the crude metabolite and its effectiveness against pathogenic bacteria, with an induced protein porin (Kumar et al., 2016). Successful attempts of epigenetic modulation have been made earlier using both the types of modifiers, i.e., 5-azacytidine as a DNMT inhibitor and sodium butyrate as a HDAC inhibitor in marine fungus *Leucostoma persoonii* for the increased production of known cytosporones B (360%), C (580%) and E (890%), and a new cytosporone R (Beau et al., 2012). Epigenetic induction of fusaric acid derivatives 5-butyl-6-oxo-1,6-dihydropyridine-2-carboxylic acid and 5-(but-9-enyl)-6-oxo-1,6-dihydropyridine-2-carboxylic acid were done using SAHA an HDAC inhibitor, in the culture medium of endophytic fungus isolated from Indian medicinal plant *Datura stramonium* (Chen et al., 2013). Three novel aromatic compounds, viz., 2′-hydroxy-6′-hydroxymethyl-4′-methylphenyl-2,6-dihydroxy-3-(2-isopentenyl) benzoate, 4,6-dihydroxy-7-hydroxymethyl-3-methylcoumarin, 4,6-dihydroxy-3,7-dimethylcoumarin and some known polyketides, endocrocin, pestalotiollide B, pestalotiopyrone G, scirpyrone A, and 7-hydroxy-2-(2-hydroxypropyl)-5-methylchromone were isolated from epigenetically induced endophytic *Pestalotiopsis acaciae* using 5-azacytidine and SAHA (Yang et al., 2013). Addition of 5-azacytidine, and/or SAHA, to the culture medium of endophytic fungus *Alternaria* sp. from *Datura stramonium* induced the production of mycotoxins, like alternariol, alternariol-5-*O*-methyl ether, 3′-hydroxyalternariol-5-*O*-methyl ether, altenusin, tenuazonic acid, and altertoxin II (Sun et al., 2012).

In a mycodiesel-producing endophyte *Hypoxylon* sp. (CI-4), 5-azacytidine treatment increased the ratio of ethanol to the total mass of all other ionizable volatile organic compounds (VOCs), from ∼0.6 to ∼0.8 while several other compounds like terpenes such as α-thujene, sabinene, γ-terpinene, α-terpinolene and β-selinene, several primary and secondary alkanes, alkenes, organic acids and derivatives of benzene that were not previously observed in the untreated culture were also observed in variable amounts with 5-azacytidine and SAHA treatments (Ul-Hassan et al., 2012). These studies clearly demonstrate that fungi like other eukaryotic microbes have genes for a wide range of potential bioactive metabolites, which remain silent/or unexpressed under normal conditions. Further, recent advancement in the next generation sequencing approaches like expression of PKS, NRPS, terpene synthase, or dimethylallyl-tryptophan synthase can be used to get the required information regarding biosynthetic gene clusters and prediction of the secondary metabolites (Cacho et al., 2014).

## Conclusion

Both, grape skin extract and turmeric extract were found effective in inducing the endophytic fungus *C*. *gloeosporioides* for isolation of bioactive cryptic metabolites and thereby increasing the antibacterial and antioxidant activities in the treated cultures. This study successfully establishes the importance of active dietary components which also interact with the epigenetic targets and can significantly induce the production of cryptic metabolites in the endophytic fungus. However, the evaluation of pure resveratrol and curcumin, the key components of the grape skin extract and turmeric extract, respectively, for their direct role in the epigenetic modulation needs to be elucidated further. The protein level changes brought out by the treatment of these extracts will further help explore the exact mechanisms/pathway involved.

## Author Contributions

Conceived and designed the experiments: VS, JK, and RK. Performed the experiments: VS, JK, NS, DS, and AM. Analyzed the data: VS, AM, SG, SV, and AK. Contributed reagents/materials/analysis tools: VS and RK. Wrote the paper: VS, JK, RK, AM, and SV.

## Conflict of Interest Statement

The authors declare that the research was conducted in the absence of any commercial or financial relationships that could be construed as a potential conflict of interest.
