# Supplementary material for: Induction of Cryptic and Bioactive Metabolites through Natural Dietary Components in an Endophytic Fungus Colletotrichum gloeosporioides (Penz.) Sacc
Source: Front Microbiol. 2017 Jun 19;8:1126. doi: 10.3389/fmicb.2017.01126 (PMC5474492; doi:10.3389/fmicb.2017.01126)
Supplement: Supplementary file 1 [file Table_1.DOCX]

Supplementary Table 1. Number of peaks detected in HPLC profiles of the total metabolites of *C*. *gloeosporioides* at wavelength of 254nm from different treatments

| **Peak No.** | **Retention Time** | **Control** | **Grape skin extract** | **Turmeric extract** |
| --- | --- | --- | --- | --- |
|  |  | (Peak area) | (Peak area) | (Peak area) |
| 1 | 0.6 | 0 | 46858 | 7957 |
| 2 | 1.1 | 3265 | 0 | 3965 |
| 3 | 1.4 | 0 | 3791 | 0 |
| 4 | 1.8 | 97245 | 7900 | 12343 |
| 5 | 2.4 | 6011 | 0 | 58060 |
| 6 | 3.1 | 846140 | 1190327 | 1170499 |
| 7 | 3.5 | 350863 | 488473 | 312994 |
| 8 | 3.7 | 0 | 0 | 270369 |
| 9 | 4.1 | 0 | 0 | 459250 |
| 10 | 4.4 | 355922 | 279860 | 0 |
| 11 | 4.8 | 0 | 145848 | 242230 |
| 12 | 5.3 | 667429 | 309329 | 582904 |
| 13 | 5.7 | 0 | 264686 | 328693 |
| 14 | 6.3 | 0 | 62897 | 0 |
| 15 | 6.4 | 543536 | 621610 | 478063 |
| 16 | 6.7 | 3986532 | 1579334 | 5175295 |
| 17 | 6.9 | 0 | 3331780 | 0 |
| 18 | 7.6 | 0 | 46276 | 0 |
| 19 | 7.9 | 16821 | 0 | 4383 |
| 20 | 8.4 | 0 | 22490 | 0 |
| 21 | 8.7 | 38717 | 0 | 0 |
| 22 | 9.4 | 1212175 | 1463103 | 1966227 |
| 23 | 10 | 516871 | 609251 | 404858 |
| 24 | 11.2 | 172519 | 454660 | 571234 |
| 25 | 12.7 | 13529 | 177448 | 42772 |
| 26 | 13.2 | 14253 | 0 | 56044 |
| 27 | 13.6 | 0 | 223193 | 0 |
| 28 | 14 | 0 | 163972 | 83993 |
| 29 | 14.5 | 0 | 0 | 37417 |
| 30 | 14.8 | 0 | 581177 | 0 |
| 31 | 15.4 | 0 | 0 | 73487 |
| 32 | 15.7 | 132245 | 0 | 0 |
| 33 | 16.4 | 354927 | 365684 | 115993 |
| 34 | 16.8 | 0 | 130884 | 0 |
| 35 | 17 | 16439 | 340537 | 0 |
| 36 | 17.4 | 279287 | 0 | 234375 |
| 37 | 17.8 | 0 | 0 | 65384 |
| 38 | 18.3 | 196873 | 0 | 475676 |
| 39 | 18.8 | 0 | 187204 | 0 |
| 40 | 19.1 | 32556 | 156327 | 0 |
| 41 | 19.4 | 56636 | 0 | 0 |
| 42 | 19.6 | 60024 | 142879 | 0 |
| 43 | 20 | 83142 | 0 | 598409 |
| 44 | 20.7 | 37176 | 0 | 0 |
| 45 | 21.1 | 0 | 270438 | 133564 |
| 46 | 21.5 | 102660 | 0 | 78594 |
| 47 | 21.8 | 0 | 0 | 71910 |
| 48 | 22.3 | 0 | 82021 | 0 |
| 49 | 22.8 | 0 | 139758 | 249417 |
| 50 | 23 | 65596 | 0 | 0 |
| 51 | 24 | 0 | 0 | 66799 |
| 52 | 24.9 | 0 | 16013 | 0 |
| 53 | 25.8 | 0 | 31580 | 0 |
| 54 | 26.7 | 12224 | 0 | 10279 |
| 55 | 27.5 | 0 | 107384 | 0 |
| 56 | 28.9 | 105936 | 0 | 89794 |
| 57 | 31.5 | 301399 | 83750 | 458402 |
| 58 | 33.7 | 0 | 245885 | 0 |
| 59 | 34.4 | 307489 | 0 | 0 |
| 60 | 36.4 | 0 | 57455 | 0 |
| 61 | 47.2 | 164859 | 0 | 0 |
| 62 | 48.7 | 12518 | 105905 | 162773 |
